# Tourette-like syndrome secondary to Kleefstra syndrome 1 with a de novo microdeletion in the *EHMT1* gene

**DOI:** 10.1186/s12883-023-03417-x

**Published:** 2023-10-10

**Authors:** Mengyue Niu, Yanjing Li, Shikun Zhan, Bomin Sun, Jun Liu, Yiwen Wu

**Affiliations:** 1 Department of Neurology, Institute of Neurology, Ruijin Hospital, Shanghai Jiao Tong University School of Medicine, Shanghai, China; 2 Department of Neurosurgery, Center for Functional Neurosurgery, Ruijin Hospital, Shanghai Jiao Tong University School of Medicine, Shanghai, China

**Keywords:** Tourette syndrome, Kleefstra syndrome 1, *EHMT1* gene, Intellectual disability, Tic disorders

## Abstract

**Background:**

Gills de la Tourette syndrome (TS) is a childhood-onset neurodevelopmental disorder manifested by motor and vocal tics. Kleefstra syndrome 1 (KS1), a rare genetic disorder, is caused by haploinsufficiency of the *EHMT1* gene and is characterized by intellectual disability (ID), childhood hypotonia, and distinctive facial features. Tourette-like syndrome in KS1 has rarely been reported.

**Case presentation:**

Here we describe a 7-year-old girl presenting involuntary motor and vocal tics, intellectual disability, childhood hypotonia, and dysmorphic craniofacial appearances, as well as comorbidities including attention deficit-hyperactivity disorder (ADHD), obsessive-compulsive disorder (OCD), and self-injurious behavior (SIB). The patient’s CNV-seq testing revealed a de novo 320-kb deletion in the 9q34.3 region encompassing the *EHMT1* gene.

**Conclusions:**

This is the first case reporting Tourette-like syndrome secondary to KS1 with a de novo microdeletion in the *EHMT1* gene. Our case suggests TS with ID and facial anomalies indicate a genetic cause and broadens the phenotypic and genotypic spectrum of both TS and KS1.

**Supplementary Information:**

The online version contains supplementary material available at 10.1186/s12883-023-03417-x.

## Background

Tourette syndrome is a chronic neurodevelopmental disorder with involuntary motor and vocal tics like eye blinking, facial grimacing, throat clearing, sniffing, or unintentional vocalizations [1]. It typically manifests in childhood and may persist into adulthood, with highly heterogeneous severities. Comorbidities, including depression, OCD, ADHD, and anxiety, commonly accompany Tourette syndrome. While its etiology remains unclear, recent research has suggested that genetic and neurochemical factors contribute to its pathogenesis [2]. KS1 is caused by either a partial deletion of the *EHMT1* gene located at chromosome 9q34.3 or a pathogenic variant within the intragenic *EHMT1* sequence [3]. With this report, we propose that KS1 be regarded as a potential etiological factor contributing to Tourette-like syndrome. Furthermore, we provide evidence supporting a de novo 320-kb deletion within the *EHMT1* gene as the causative genetic aberration underlying this particular manifestation of KS1.

## Case presentation

A 7-year-old girl presented with an intellectual disability and four years of tic disorders. She was born to nonconsanguineous parents with unremarkable family history. Auxological parameters at birth were within the normal range. Her motor and language developmental milestones were delayed. At the age of 9 months, she was unable to turn, crawl or sit independently. Clinical and neurological examination revealed hypotonia, reduced movements, funnel chest, and hyperextended knees. The audiometric evaluation showed bilateral hearing impairment. The patient could not walk or speak single words until age 2. She underwent intensive speech training with slow improvement. At age 3, she developed involuntary bursts of screaming and has since experienced eye blinking, facial grimacing, and shoulder shrugging (Video S1). A diagnosis of TS was established one year later. Risperidone was initially tried without significant improvements. Aripiprazole and clonazepam were added, relieving her motor tics for a short duration. In the subsequent years, she developed several comorbidities, including attention deficit-hyperactivity disorder (ADHD), obsessive-compulsive disorder (OCD) and self-injurious behavior (SIB) for the presence of inattention, hyperactivity, cleaning rituals, rage attacks, picking at the finger skin to bleeding and repetitive slapping on the head (Video S1). The patient went to special education school due to learning difficulties.

**Fig. 1:**
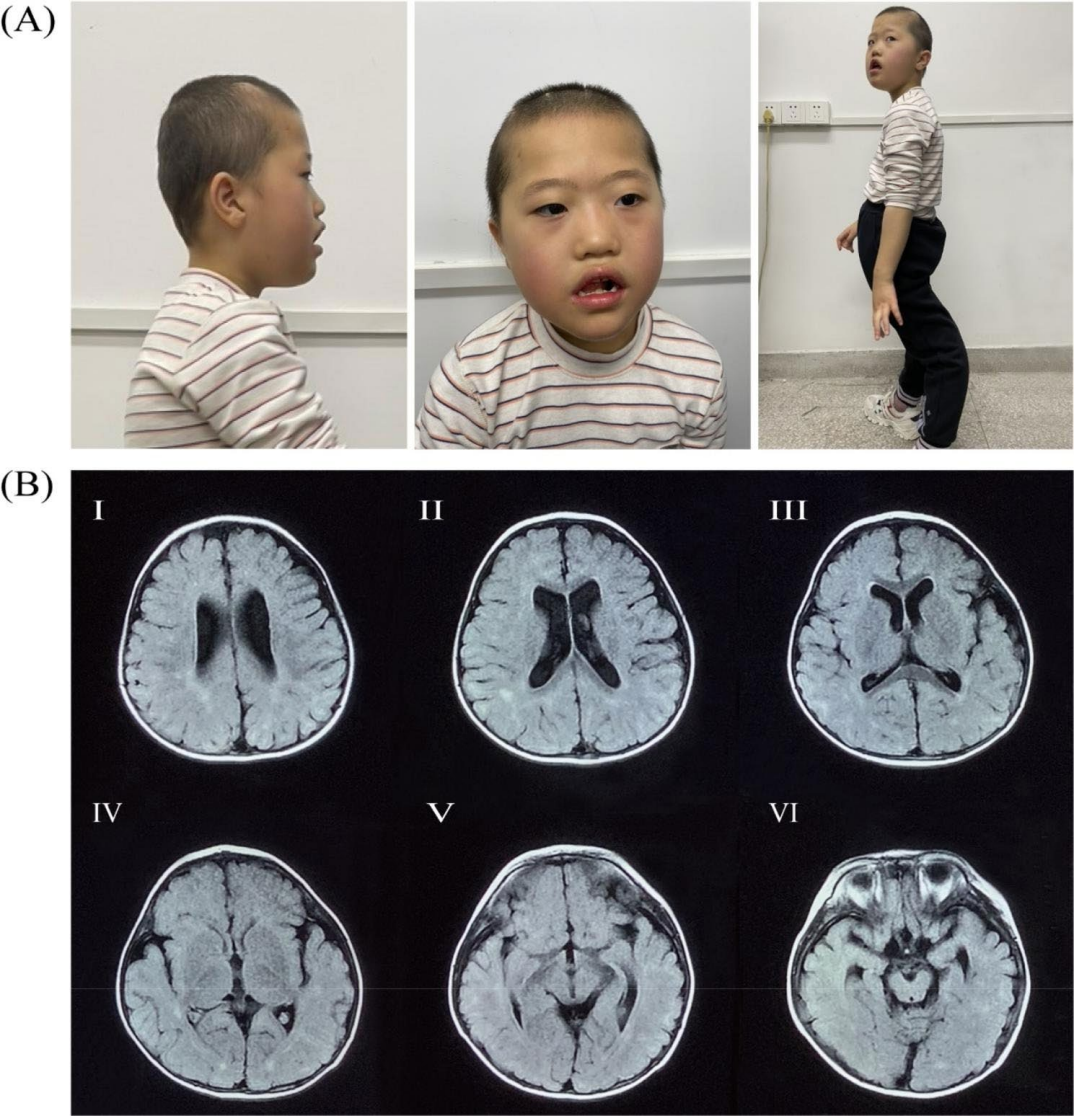
Facial appearance and brain MRI of the patient. (A) Typical KS1 facial features and hyperextended knees in our patient. (B) Axial T2 Flair images showing white matter hyperintensities

Physical examination revealed dysmorphic craniofacial appearance including brachycephaly, broad forehead, arched eyebrows, epicanthal folds, midface retrusion, low-set ears with thickened helices, everted lower lip and tented upper lip as well as hyperextended knees (Fig. 1A). Insuppressible motor and vocal tics were frequently observed. The patient could only answer a few simple questions with slurred speech. Routine laboratory tests, including metabolic and genetic screening (whole-exome sequencing), were unremarkable. Brain MRI revealed periventricular white matter hyperintensities (Fig. 1B). Suspecting KS1 due to the typical facial features, CNV-seq was performed for the family after written consent. The parents’ CNV-seq screening was negative. The patient demonstrated a de novo 320-kb deletion in the 9q34.3 region (chr9:140721998–141,041,998) encompassing the *EHMT1* gene.

## Discussion and conclusion

Although this patient had both motor and vocal tics for more than one year with childhood onset, primary TS was not considered. She did not exhibit a waxing-waning course and had a general medical condition as KS1. KS1 is genetically and phenotypically heterogeneous with multisystem dysfunction [3]. KS1 is characterized by a variable genotype-phenotype correlation, mainly owing to its heterogeneous genetic background. Both deletions and intragenic variants of the *EHMT1* gene can result in the KS1. The manifestations of KS1 are primarily attributed to the loss of function of the *EHMT1* gene. Individuals harboring intragenic pathogenic variants of the *EHMT1* gene (e.g., missense or frameshift mutations) or those with smaller deletions (less than 1 Mb) in the 9q34.3 region typically exhibit comparable phenotypic characteristics. However, those with larger deletions (≥ 1 Mb) tend to present more severe intellectual disability and additional medical complications, such as congenital anomalies and feeding issues [3]. Upon literature review, approximately 120 cases with various *EHMT1* gene mutations have been reported to date [4]. The distinctive facial features of KS1 are characterized by brachy(-micro)cephaly, broad forehead, unusual shape of eyebrows (arched or straight with synophrys), mildly upslanted palpebral fissures, midface retrusion, thickened ear helices, short nose with anteverted nares, fleshy everted vermilion of the lower lip and exaggerated cupid’s bow or “tented” appearance of the vermilion of the upper lip, and protruding tongue and relative prognathism [3]. The craniofacial characteristics of this patient are closely aligned with the facial presentation typically associated with KS1. Other suggestive clinical findings include intellectual disability, childhood hypotonia, motor delay, hearing loss, and ASD. Apart from *EHMT1*, the de novo 320-kb deletion in the 9q34.3 region also affects the *CACNA1B* gene, which is associated with Dystonia 23 (DYT23). DYT23 is characterized by adult-onset, non-progressive, focal cervical dystonia, none of which were observed in our patient. Therefore, despite the deletion’s impact on the *CACNA1B* gene, the lack of corresponding phenotypic evidence leads us to focus our interpretation on the effect of the *EHMT1* gene deletion in this case.

Tic disorders were rarely reported in KS1. Only one case has described a 24-year-old KS1 patient with childhood hypotonia, developmental delay, adolescent onset vocal tics (coprolalia), OCD, and ASD. The authors demonstrated that the strategic positioning of deep brain stimulation (DBS) leads within the bilateral ventral capsule/ventral striatum region led to a gradual and sustained amelioration of the patient’s compulsive behaviors, coprolalia, and social interaction [5]. DBS has shown promise as a neurosurgical treatment for selected individuals with severe, treatment-refractory Tourette syndrome, and its safety profile is generally favorable when executed by an experienced multidisciplinary team. Numerous open-label and retrospective studies, along with multicenter retrospective studies and meta-analyses, have reported significant improvements in tics and neuropsychiatric symptoms following DBS [2]. These suggest that targeted DBS holds promise as a viable therapeutic option for KS1 patients with intractable tics and other neuropsychiatric disorders. Further research and case studies are needed to provide more support for this treatment approach in the KS1 population.

Research over the past few decades has consistently shown that genetics plays a significant role in developing TS [2]. The exact genetic mechanism underlying TS has yet to be fully understood. Still, it involves multiple genes that regulate neurotransmitters and synaptic plasticity [6, 7]. Intriguingly, common TS comorbidities, including OCD, ADHD, and ASD, have been reported in KS1 patients, suggesting a shared genetic and neurobiological basis [8–10]. The *EHMT1* gene encodes for Euchromatic Histone Methyltransferase-1, which, in conjunction with *EHMT2*, is responsible for the dimethylation of ‘Lys-9’ (H3K9me2) on histone H3—a process vital to chromatin organization and gene expression. Research has shown that *EHMT1* plays an essential role in homeostatic synaptic scaling up [11]. Synaptic scaling is a well-characterized form of homeostatic synaptic plasticity that maintains the stability of neuronal network activity by striking a balance between excitation and inhibition. Notably, aberrations in synaptic plasticity are observed in TS [12]. Therefore, it is plausible to suggest that *EHMT1* haploinsufficiency could interfere with normal synaptic scaling up, thereby potentially contributing to the pathogenesis of TS. However, the exact mechanisms by which EHMT1 mutations may lead to TS remain to be elucidated. Further research is needed to fully comprehend the complexities of these interactions. In conclusion, our case suggests TS with ID and facial anomalies indicate a genetic cause and expands the phenotypic and genotypic spectrum of both TS and KS1.

### Electronic supplementary material

Below is the link to the electronic supplementary material.

Supplementary Material 1

Supplementary Material 2

## Data Availability

The data supporting this case report’s findings are available on request from the corresponding author.
