# Supplementary material for: Tourette-like syndrome secondary to Kleefstra syndrome 1 with a de novo microdeletion in the EHMT1 gene
Source: BMC Neurol. 2023 Oct 10;23:365. doi: 10.1186/s12883-023-03417-x (PMC10563308; doi:10.1186/s12883-023-03417-x)
Supplement: Supplementary file 2 — Supplementary Material 2 [file 12883_2023_3417_MOESM2_ESM.docx]

**Video S1.** Vocal and motor tics in our patient.

At age 3, the patient developed involuntary bursts of screaming. In the subsequent years, the patient experienced eye blinking, facial grimacing, and shoulder shrugging. Inattention, hyperactivity, and repetitive head slapping were also noticed in the patient.
